# Gold Nanoparticles Combined Human β-Defensin 3 Gene-Modified Human Periodontal Ligament Cells Alleviate Periodontal Destruction *via* the p38 MAPK Pathway

**DOI:** 10.3389/fbioe.2021.631191

**Published:** 2021-01-28

**Authors:** Lingjun Li, Yangheng Zhang, Min Wang, Jing Zhou, Qian Zhang, Wenrong Yang, Yanfen Li, Fuhua Yan

**Affiliations:** ^1^Nanjing Stomatological Hospital, Medical School of Nanjing University, Nanjing, China; ^2^Key Laboratory of Oral Biomedical Research of Zhejiang Province, The Affiliated Stomatological Hospital, Zhejiang University School of Medicine, Zhejiang University School of Stomatology, Hangzhou, China; ^3^School of Life and Environmental Science, Centre for Chemistry and Biotechnology, Deakin University, Geelong, VIC, Australia

**Keywords:** gold nanoparticles, human β-defensin 3 gene, osteogenic differentiation, p38 MAPK pathway, periodontal ligament cells

## Abstract

Periodontitis is a chronic inflammatory disease with plaques as the initiating factor, which will induce the destruction of periodontal tissues. Numerous studies focused on how to obtain periodontal tissue regeneration in inflammatory environments. Previous studies have reported adenovirus-mediated human β-defensin 3 (hBD3) gene transfer could potentially enhance the osteogenic differentiation of human periodontal ligament cells (hPDLCs) and bone repair in periodontitis. Gold nanoparticles (AuNPs), the ideal inorganic nanomaterials in biomedicine applications, were proved to have synergetic effects with gene transfection. To further observe the potential promoting effects, AuNPs were added to the transfected cells. The results showed the positive effects of osteogenic differentiation while applying AuNPs into hPDLCs transfected by adenovirus encoding hBD3 gene. *In vivo*, after rat periodontal ligament cell (rPDLC) transplantation into SD rats with periodontitis, AuNPs combined hBD3 gene modification could also promote periodontal regeneration. The p38 mitogen-activated protein kinase (MAPK) pathway was demonstrated to potentially regulate both the *in vitro* and *in vivo* processes. In conclusion, AuNPs can promote the osteogenic differentiation of hBD3 gene-modified hPDLCs and periodontal regeneration *via* the p38 MAPK pathway.

## Introduction

Inflammatory responses and bone loss occurring in periodontitis have become the most critical and challenging problem to be solved to achieve healthy periodontal tissues (Taut et al., 2013; Bassir et al., 2016). Human β-defensin 3 (hBD3), a small molecule cationic antimicrobial peptide consisting of 45 amino acids, is perceived to be the most promising antimicrobial peptide (Harder et al., 2001; Dhople et al., 2006). Former studies have demonstrated that hBD3 is perceived to be the most promising antimicrobial peptide and can potentially promote the osteogenic differentiation of human periodontal ligament cells (hPDLCs) in inflammatory microenvironments (Zhou et al., 2018). However, recombinant hBD3 is extremely expensive and difficult to store. Therefore, our team previously synthesized adenovirus vectors containing the hBD3 gene and successfully transfected them into hPDLCs. The enhancement effects of osteogenic differentiation and bone regeneration have also been observed after hBD3 gene transfection (Li et al., 2020).

Gold nanoparticles (AuNPs) are recognized as ideal inorganic nanomaterials concerning biomedicine applications, including drug delivery, diagnostic imaging, and targeting therapy, due to their excellent biocompatibility and versatility in surface modification (Dreaden et al., 2012; Hu et al., 2020). For decades, nanotechnology, such as nanofabrication, nanofiltration, and synergistic therapy, has revealed an inextricable relationship with viral biology. A study in 2003 showed that AuNPs can be spectroscopic enhancers by covering viruses with 2.5–4.5 nm AuNPs (Dragnea et al., 2003). Guo et al. (2010) demonstrated the feasibility of using charge-reversal functional AuNPs as a means of improving the nucleic acid delivery efficiency. Another study proved that gold-based nanoparticles complexed with exogenous pDNA improved transfection efficiency in human mesenchymal stem cells (Yi et al., 2019). Hence, we supposed that AuNPs could also promote hBD3 gene transfection in PDLCs thereby promoting osteogenesis and bone regeneration.

The process of osteogenic differentiation involves the activation of multiple signaling pathways (Long et al., 2019; Ohashi et al., 2019). Among these, the p38 MAPK pathway is one of the critical pathways; it has been reported to regulate the osteogenesis process in various cells, including hPDLCs (Yi et al., 2010; Ren et al., 2013; Li et al., 2020). There are few reports about hBD3 and osteogenesis, yet other cationic antimicrobial peptides like LL37 were demonstrated to affect the proliferation and differentiation of MC3T3-E1 cells (Shen et al., 2019) and could enhance bone regeneration in a rat calvarial bone defect through p38 MAPK pathways (Kittaka et al., 2013).

Hence, we aim to evaluate the role of AuNPs in the osteogenic differentiation process of hBD3 gene transfected hPDLCs in inflammatory microenvironments. Further experiments were conducted to explore the potential mechanism underlying these phenomena. SD rat periodontal ligament cells (rPDLCs) transfected with the hBD3 gene and AuNPs were transplanted into rats with periodontitis to observe the effects of AuNPs *in vivo*.

## Materials and Methods

We declared that all experiments adhered to standard biosecurity and institutional safety procedures. For *in vitro* experiments, at least four replicate wells per group were set up, and an average of six SD rats per group were used for animal experiments.

### Gold Nanoparticle Characterization

Gold nanoparticles with diameters of 45 nm were synthesized at the School of Life and Environmental Science, Centre for Chemistry and Biotechnology, Deakin University. The concentration of the gold colloid solution was approximately 0.25 mM. The next step was adding 100 μL of 0.1 mM L-cysteine (Sigma-Aldrich, St. Louis, MO, United States) solution into every 5 mL of AuNPs with stirring for 2 h. The AuNPs were rinsed by centrifugation to remove unbound L-cysteine and dispersed in deionized water (Kalmodia et al., 2013; Weng et al., 2013; Zhang et al., 2017). Our team has previously demonstrated that L-cysteine-modified AuNPs of 45 nm and 10 μM are appropriate and biocompatible when applied to hPDLCs (Zhang et al., 2017).

### Cell Culture and Gene Transfection

Human periodontal ligament cells from healthy donors were obtained from ScienCell (ScienCell Research Laboratories, San Diego, CA, United States). The cells were cultured in Dulbecco’s modified Eagle’s medium (DMEM, Gibco, Grand Island, NY, United States) with 1% penicillin/streptomycin (Gibco, Grand Island, NY, United States) and 10% fetal bovine serum (FBS, ScienCell Research Laboratories, San Diego, CA, United States) at 37°C in 5% CO_2_. Every other day, the medium was refreshed, and cells between passages 2 and 6 were used. Osteogenic differentiation medium (growth medium supplemented with 50 μg/mL ascorbic acid (Sigma-Aldrich, St. Louis, MO, United States), 10 mM β-glycerophosphate (Sigma-Aldrich, St. Louis, MO, United States), and 100 nM dexamethasone (Sigma-Aldrich, St. Louis, MO, United States) was used to replace the original growth medium every 2 days.

The recombinant adenovirus carried the human beta defensin-3 gene (Ad-hBD3) was purchased from the GenePharma company (GenePharma, Shanghai, China). The hPDLCs were transfected with Ad-hBD3 according to our former study (Li et al., 2020).

### Quantitative Real-Time PCR

The mRNA levels of hBD3 and osteogenesis-related genes were determined by Quantitative Real-Time PCR (qRT-PCR). Six-well plates with growth medium were chosen to seed hPDLCs (1 × 10^5^ cells/well). The transfections of Ad-hBD3 were conducted when the cell fusion rate reached 80%. On day 3, after hBD3 expression was identified, the medium with AuNPs (45 nm, 10 μM) was replaced for the next experiment. *Escherichia coli*-LPS (1 μg/mL) was added into the medium 2 h later, ensuring that the AuNPs were already taken in by the cells (Alkilany and Murphy, 2010). TRNzol reagent (Tiangen, Beijing, China) was adopted to extract total RNA from the cells. The reverse transcription of total RNA to cDNA was performed by using the PrimeScript RT Reagent Kit (Takara, Otsu, Japan). The sequences of the primers for qRT-PCR are shown in Table 1. Each gene cycle threshold (ct) was normalized based on the ct of GAPDH examined simultaneously on the same plate and then calculated by the comparative 2^−ΔΔCt^ method. All of the samples were run in triplicate.

**TABLE 1:** Primer sequences.

Primer name	Forward primer sequence (5′–3′)	Reverse primer sequence (5′–3′)
GAPDH	GGCGTGATGGCTTATTTGTT	GGCGTGATGGCTTATTTGTT
Runx2	AACCCACGAATGCACTATCCA	CGGACATACCGAGGGACATG
COL1	CTGCAAGAACAGCATTGCAT	GGCGTGATGGCTTATTTGTT
ALP	CCGTGGCAACTCTATCTTTGG	GCCATACAGGATGGCAGTGA
hBD3	AAGCCTAGCAGCTATGAGGATCC	TGTGTTTATGATTCCTCCATGACC

### Western Blot

The expression levels of hBD3, osteogenesis-related, p38, and p-p38 proteins were measured by WB assay. Cells were lysed in RIPA buffer (Beyotime, Shanghai, China). The total proteins were denatured by boiling for 5 min, resolved by 10% gradient sodium dodecyl sulfate-polyacrylamide gel electrophoresis and then transferred onto polyvinylidene difluoride membranes (Millipore, Bedford, MA, United States). Five percent skim milk powder was used for blocking; 2 h later, the membranes were incubated overnight with primary antibodies, anti-hBD3 (ab19270), anti- ALP (ab83259), anti-Runx2 (ab23981), anti-COL1 (ab96723) (Abcam, Cambridge, United Kingdom), anti-p38 (#8690 S), and anti-p-p38 (#4511 S) (CST, MA, United States), with anti-β-actin (BS6008M) (Bioworld, MN, United States) as the housekeeping gene, at 4°C, and then antirabbit or anti-mouse secondary antibody was added and incubated for 1 h. A Tanon 5200 chemiluminescent imaging system (Tanon, Shanghai, China) was utilized to visualize the proteins. Image J software was used to quantify images of western blot bands. After converting the image western blot bands into a grayscale picture, we eliminated background influence and then set quantitative parameters and units to quantify. The results were exported from Image J and analyzed by graphpad prism 6.

### Transmission Electron Microscopy

Transmission electron microscopy (TEM) was used to examine the uptake of the AuNPs. hPDLCs (1 × 10^5^ cells/well) were seeded into six-well plates and cultured in a growth medium overnight. Then, the osteogenic differentiation medium containing modified AuNPs (45 nm, 10 μM) was prepared to replace the former medium. After culturing for 7 days, the cells were fixed with 2.5% glutaraldehyde, and subsequently, in 1% osmium tetroxide, dehydrated in a graded series of ethanol and ultimately embedded in epoxy resin. Ultrathin sections were examined with TEM (HT7700, Hitachi Company, Tokyo, Japan) at an accelerating voltage of 120 kV.

### Alkaline Phosphatase Activity and Staining Assay

The alkaline phosphatase (ALP) assay kit (Abcam, MA, United States) was used to assess the ALP activity. After transfection, hPDLCs (3.0 × 10^4^ cells/well) were seeded into 24-well plates. The medium was changed into an osteogenic differentiation medium with AuNPs (45 nm, 10 μM) after culturing overnight. After culturing for 7 days, the cells were rinsed twice with PBS. After a series of steps following the manufacturer’s instructions, the final solution was added to the plates. The absorbance was assessed at 405 nm by a SpectraMax M3 microplate reader (Molecular Devices, Sunnyvale, CA, United States). The ALP activity level was defined relative to the control group as a percentage after measuring a standard curve. The measurement of ALP staining was conducted on the same day according to the manufacturer’s instructions (BCIP/NBT ALP staining kit, Beyotime Institute of Biotechnology, Shanghai, China).

### ARS Staining

After cultured for 3 weeks, the cells were rinsed twice with PBS and fixed in 4% paraformaldehyde for 30 min. The cells were washed with distilled water (DW), treated with 2% alizarin red S solution (Sigma-Aldrich, United States) for 5 min, and then washed 3–5 times with DW to remove the unbound alizarin red S (ARS). For the quantification of alizarin red S staining, cells were desorbed with 10% (w/v) cetylpyridinium chloride (Sigma-Aldrich, St. Louis, MO, United States); then, the absorbance was measured at 562 nm by a SpectraMax M3 microplate reader. The stained plates were air-dried and examined under a light microscope (Olympus IMT-2, Tokyo, Japan) and photographed with a digital camera (Canon EOS 550D, Tokyo, Japan).

### Culture and Identification of rPDLCs

Female Sprague–Dawley Rats (5 weeks old) were sacrificed and then their six molars were extracted and placed into 0.1% collagenase I solution for shaking for 2 h at 37°C. After centrifugation, cells were resuspended in growth medium at 37°C in 5% CO_2_ for 3–5 days when cell adherence was observed. Afterward, the refresh frequency of the culture medium was every 2 days. After 1–2 passages, cells were fixed by 4% paraformaldehyde for the subsequent immunofluorescence staining of anti-cytokeratin and anti-vimentin (CST, MA, United States), which were used to identify the cell origin. Cells were seeded into 96-well plates (5.0 × 10^3^ cells/well), cultured for 10 days while adding 10 μL/well reagents of the cell counting kit-8 (CCK8) each day, and then incubated at 37°C in 5% CO_2_ for 4 h. The absorbance was measured with a SpectraMax M3 microplate reader (Molecular Devices, Sunnyvale, CA, United States) at a wavelength of 450 nm. Cell viability was expressed as the relative absorbance after excluding the background absorbance.

### Experimental Periodontitis and rPDLCs Transplantation

Female Sprague–Dawley Rats (5 weeks old) were randomly allocated into different groups (*N* = 6 experimental rats per group): a blank group (without ligature and cell transplantation), control group (with ligature alone), Ad-hBD3 group (with ligature and cell transplantation treated with Ad-hBD3), and Ad-hBD3 + AuNPs group (with ligature and cell transplantation treated with Ad-hBD3 and AuNPs). Silk threads soaked in a bacterium solution of *Porphyromonas gingivalis* ahead for 2 h were used to ligature the bilateral maxillary second molars of the SD rats. rPDLCs with different treatments were dissociated into cell suspension with 0.9% NaCl (1 × 10^4^ cells/μL) and then injected by a 100-μL microsyringe (Hamilton, Bonaduz, Switzerland) at the mesial, middle, and distal sites of palatal gingival tissues around the ligatured molars. After 2 and 24 h, relevant gingival tissues were gathered to make frozen sections to confirm the rPDLC transplantation. rPDLC transplantations were performed once per week, and 2 weeks later, all rats were sacrificed and sampled.

### Micro-CT Analysis

The maxillary bone samples of the SD rats were trimmed and placed into 4% paraformaldehyde fixative solution for 24 h. On the next day, the samples were collected and prepared for micro-CT scanning with a Skyscan 1176 (Bruker, Karlsruhe, Germany). The scanning parameters were as follows: a rotation angle of 360°, a tube voltage of 70 kV, a tube current of 353 μA, an X-ray exposure time of 404 ms, and a scanning layer thickness of 18 μm. The data were reconstructed with NRecon software and then imported into CTVox and CTAn software to obtain three-dimensional (3D) images and relative data. We selected the alveolar bone area around the root of the second molar form alveolar bone crest to apical as the ROI. There were almost 100 slides in different groups.

### Histological Analysis

Two weeks later, the SD rats were sacrificed by euthanasia. The right maxillas were collected to be fixed in 4% paraformaldehyde for 48 h and then placed into 10% EDTA decalcifying solution for 1 month. Afterward, the specimens were dehydrated with a gradient series of 40, 50, 60, 70, 80, 90, and 95% ethanol for 12 h each, and they were finally soaked in a 95% ethanol and xylene solution mixture (1:1) for 12 h to make them transparent. After sectioning the samples to a thickness of 5 μm, they were stained with a p-p38 antibody, and the mean density of p-p38 was measured in three different samples.

### Statistical Analysis

All statistical calculations were performed with SPSS23 statistical software (SPSS, Chicago, IL, United States). Quantitative data were expressed as the mean ± standard deviation (SD). Significant differences were determined using Student’s t-test (for normally distributed samples where the total sample content is small (e.g., n < 30), the overall standard deviation σ is unknown) or one-way ANOVA (comparison of population means corresponding to three or more groups of samples) followed by the Bonferroni test. Statistical significance was determined by different tests depending on the situation and was considered statistically significant at p-values of less than 0.05.

## Results

### Cellular Uptake and Localization of AuNPs

**FIGURE 1:**
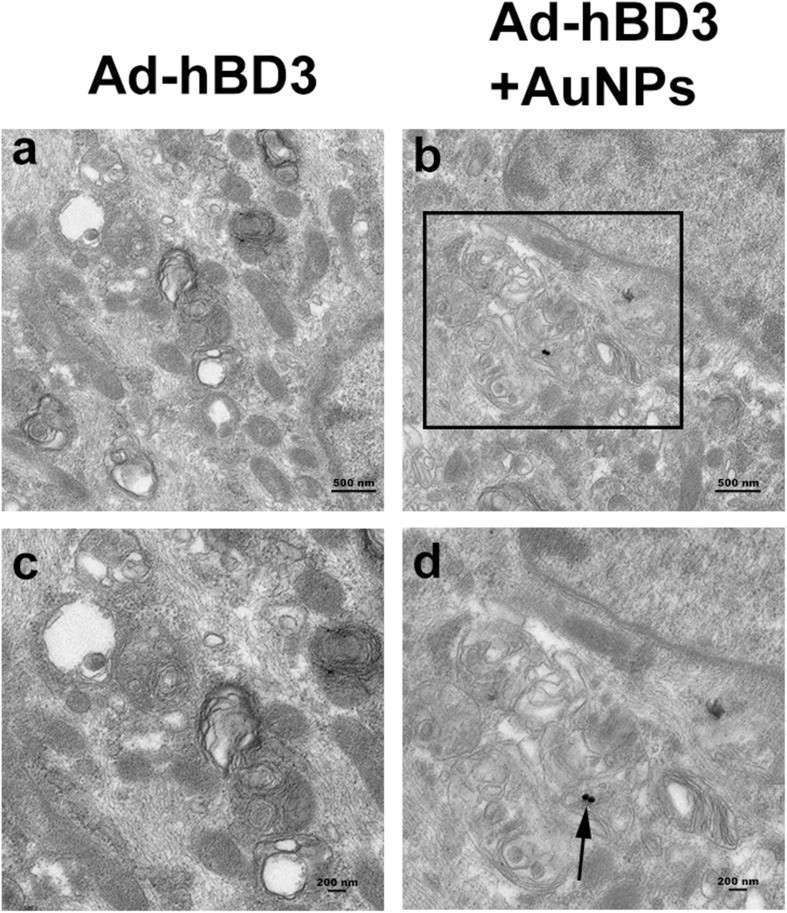
Cellular uptake of AuNPs in hPDLCs. hPDLCs were transfected by Ad-hBD3 and treated with AuNPs (45 nm, 10 μM) depending on the grouping. **(A)** TEM images of groups on day 7 without AuNPs. **(B)** TEM images of groups on day 7 with AuNPs. Panels **(C,D)** were high power views of the indicated portion in panels **(A,B)** separately. Arrows indicate internalized AuNPs.

After AuNPs (45 nm, 10 μM) treatment and cultured for 7 days, the ingestion and positioning of AuNPs in hPDLCs were detected by TEM. Figure 1 shown that AuNPs taken up by cells were generally found in intracellular vesicles. We could also observe one or two AuNPs clustered together in some cells. For the most part, AuNPS were detected in cellular organelles including lysosome and autophagosome.

### Osteogenic Enhancement of AuNPs on Transfected hPDLCs

**FIGURE 2:**
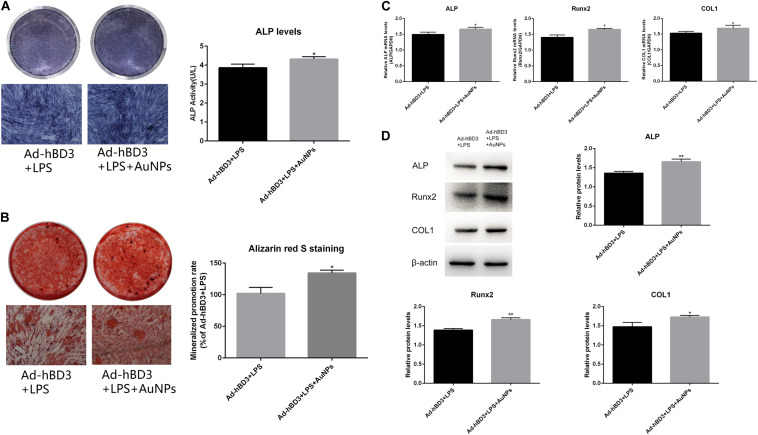
ALP and ARS staining assays of hPDLCs. **(A)** ALP staining and activity on day 7, **(B)** Alizarin red S staining of mineralized nodules on day 21. Osteogenesis-related gene and protein expression. hPDLCs were transfected by Ad-hBD3 and treated with AuNPs. *E. coli*-LPS was added in all groups to create inflammatory microenvironments. **(C)** qRT-PCR and **(D)** western blot analysis of ALP, Runx2, and COL1 gene and protein expressions on day 7 (*p < 0.05 and **p < 0.01).

The results of ALP and ARS staining revealed that the ALP staining color was darker in the Ad-hBD3 + AuNPs groups than that in the Ad-hBD3 groups. The number of mineral nodules was also higher in the Ad-hBD3 + AuNPs groups (Figures 2A,B). qRT-PCR and western blot assay were conducted on day 7. The results showed that ALP, Runx2, and COL1 expressions were significantly higher in AuNP-treated groups than in AuNP-free groups at both the mRNA and protein levels (Figures 2C,D).

### Activation of p38 MAPK Pathway *in vitro*

**FIGURE 3:**
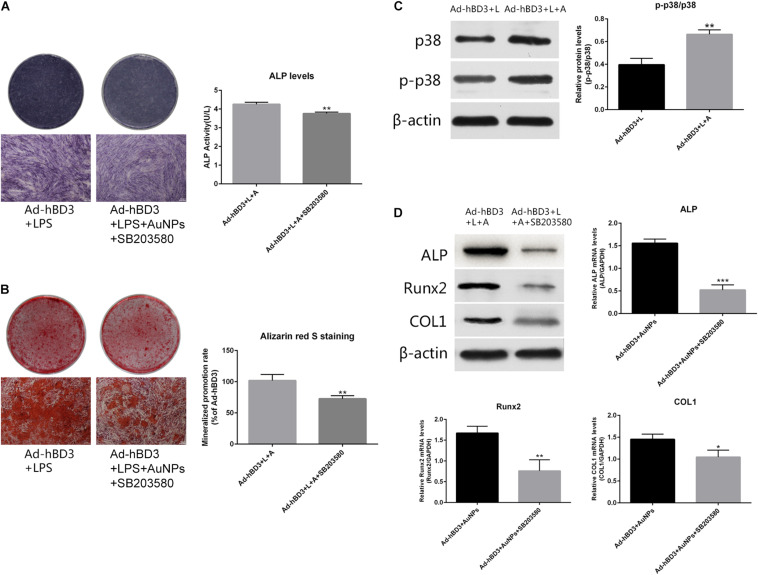
Effects of AuNPs on p38 MAPK pathway activation in hBD3 gene transfected hPDLCs. **(A)** ALP staining and ALP activity on day 7 after adding SB203580; **(B)** Alizarin red S staining of mineralized nodules on day 21 after adding SB203580; **(C)** High expression of p-p38 was observed in Ad-hBD3 + AuNPs groups; **(D)** Bone-related gene and protein expressions after adding SB203580. Groups treated with the inhibitor showed low expression of ALP, Runx2, and COL1 (*p < 0.05, **p < 0.01, and ***p < 0.001).

To investigate the potential mechanism of the osteogenesis-promoting effects of AuNP on hBD3 gene transfected hPDLCs in inflammatory microenvironments, the following experiments were conducted. On day 7, the expression of p-p38 as the specific protein in the p38 MAPK pathway was extremely high in Ad-hBD3 + AuNPs groups (Figure 3C), which demonstrated that the p38 MAPK pathway was activated. Besides, after adding the p38 MAPK pathway inhibitor, SB203580, into the hPDLCs, ALP, Runx2, and COL1 expression decreased significantly both at the mRNA and protein level, which proved that the expression of osteogenesis-related genes and proteins could be regulated by the p38 MAPK pathway (Figure 3D). ALP on day 7 and ARS stainings on day 21 showed a similar trend, which means that the p38 MAPK pathway might still regulate the subsequent osteogenic differentiation process (Figures 3A,B).

### Primary Culture and Transplantation of rPDLC

**FIGURE 4:**
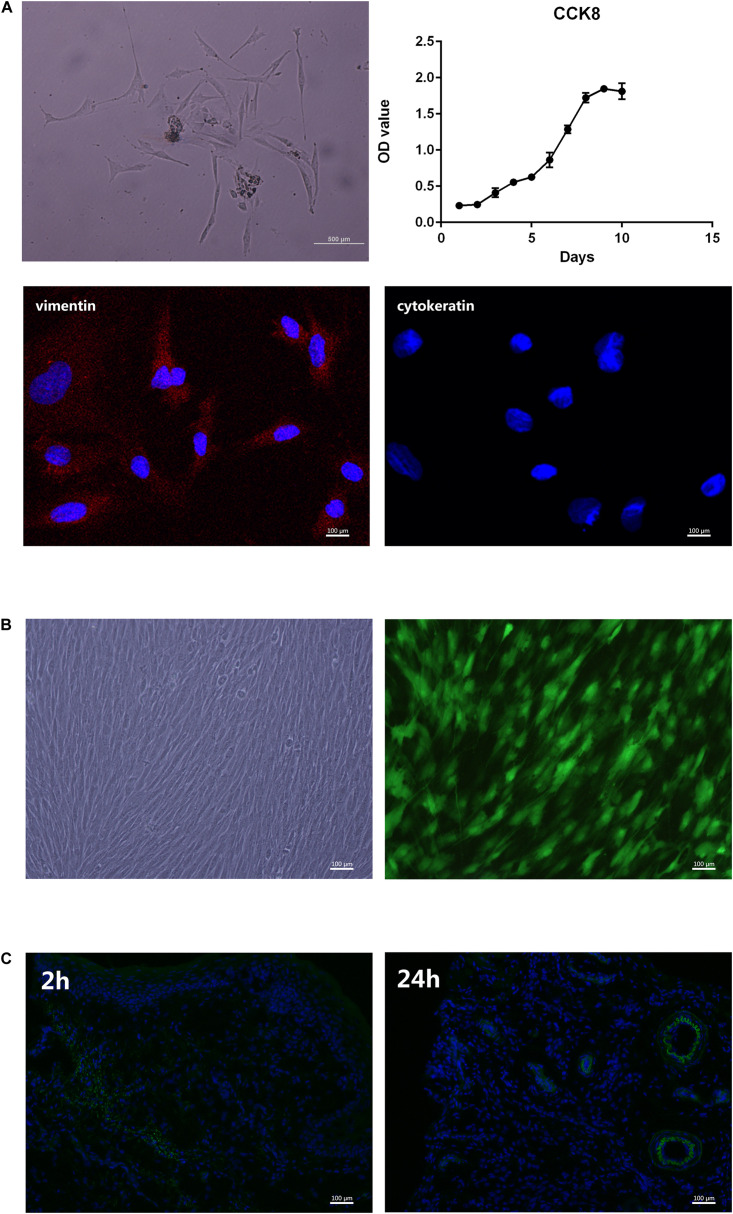
Primary culture and transplantation of rPDLC. **(A)** Characterization of rPDLCs: Cells isolated from periodontal ligament tissues of SD rats; growth curve of cultured cells; positive expression of vimentin (red); and negative expression of cytokeratin; **(B)** Fluorescence images of rPDLCs after transfection; **(C)** immunofluorescence images at 2 and 24 h after cell injection.

Figure 4A presents the cells isolated from the periodontal ligament tissues of SD rats. Immunofluorescence results showed that the cultured cells could express vimentin (red) but not cytokeratin, which indicates that they were mesoderm-derived. The three cell growth phases, lag, log (exponential), and plateau, were observed from the cell growth curve measured by CCK8. Our team has already proved that rPDLCs transfected with Ad-hBD3 could successfully express hBD3 (Li et al., 2020). Fluorescence images showed rPDLCs transfected with Ad-hBD3 after cultured for 3 days (Figure 4B). Figure 4C showed immunofluorescence images at 2 and 24 h after cell injection. The transplanted cells were diffusely distributed within the tissue at 2 h and after 24 h were more clustered around blood vessels.

### Promotion of Periodontal Regeneration After Transplantation of Ad-hBD3 Transfected rPDLC Treated With AuNPs

**FIGURE 5:**
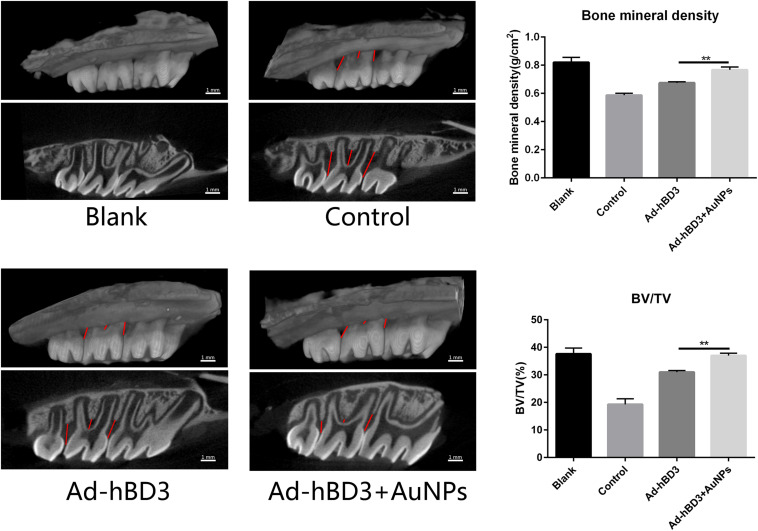
Three-dimensional reconstruction images, bone mineral density (BMD), and the ratio of bone volume to tissue volume (BT/TV). Ad-hBD3 + AuNPs groups showed less bone loss and higher BMD and BV/TV than Ad-hBD3 groups (**p < 0.01).

The SD rats were sacrificed after 2 weeks, and the bilateral maxillary bone was sampled for micro-CT scanning. The following 3D images show that the alveolar bone loss around the ligatured molars of the control group was much more obvious than that of other groups, which means that we created experimental periodontitis successfully. Also, Ad-hBD3, and Ad-hBD3 + AuNP groups presented less bone resorption and a higher bone mineral density and bone volume ratio (Figure 5). The results indicated that AuNPs can reduce bone destruction and potentially promote bone regeneration in SD rats with periodontitis after transplantation of Ad-hBD3 transfected rPDLC.

**FIGURE 6:**
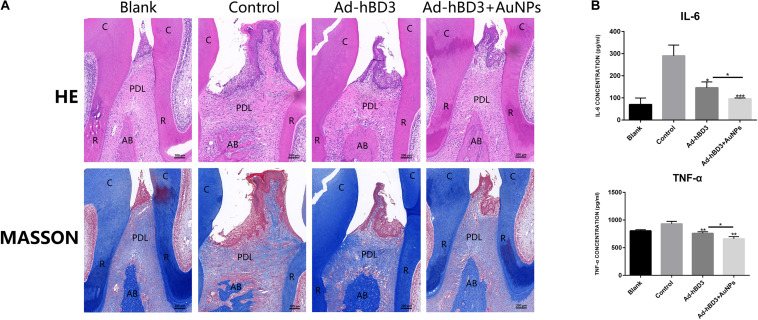
H&E, Masson’s trichrome stainings of ligatured areas and expression of inflammatory cytokine in serum. **(A)** Ad-hBD3 + AuNPs groups showed alleviative gingival inflammation and bone loss than Ad-hBD3 groups. **(B)** Ad-hBD3 + AuNPs groups showed less expression of IL-6 and TNF-α. (original magnification: ×50; scale bar 200 μm; C, crown; R, root; AB, alveolar bone; PDL, periodontal ligament; (*p < 0.05, **p < 0.01, and ***p < 0.001).

H&E and Masson’s trichrome staining results showed that in the control group, the alveolar bone around the second molar was resorbed obviously, the hyperplasia of gingival epithelial spikes and thickened stratum spinosum could be observed, as was the inflammatory cells infiltration and collagen fibers destruction. In Ad-hBD3 + AuNPs group where hBD3-rPDLCs were treated with AuNPs, we observed less inflammatory cells along with lighter epithelial spikes hyperplasia in the gingival epithelium, and the collagen fibers arranged more orderly in Ad-hBD3 + AuNPs group than the control group. Furthermore, altered and broken collagen fibers could be repaired in those groups (Figure 6A). Besides, the inflammatory cytokines of IL6, TNF-α, and IFN-γ in serum were also detected by ELISA assay (Figure 6B).

### Activation of p38 MAPK Pathway *in vivo*

**FIGURE 7:**
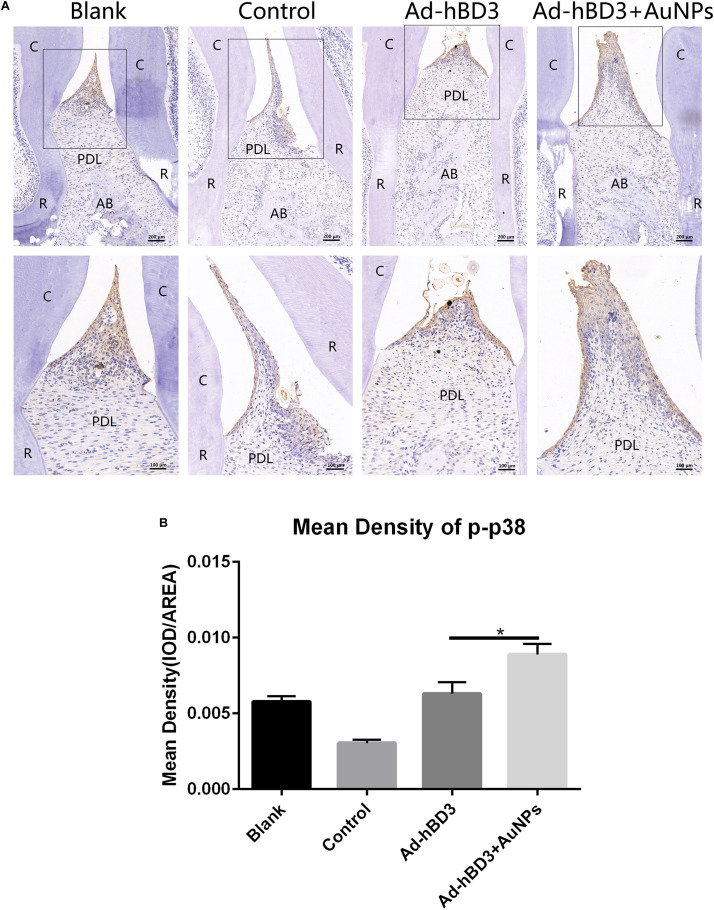
p-p38 staining and mean density (*p < 0.05). **(A)** Positive expression of p-p38 in the different groups; **(B)** mean density of p-p38 expression in the different groups (original magnification of a: ×100 and ×200; scale bar 100 μm and 50 μm; R, root; AB, alveolar bone; PDL, periodontal ligament).

Immunohistochemistry experiments of p-p38 were conducted to evaluate whether the p38 MAPK pathway was involved while applied *in vivo*. The results showed that the p-p38 expression level in Ad-hBD3 + AuNPs groups was higher than that in Ad-hBD3 groups and the expression levels of p-p38 in the Ad-hBD3 + AuNPs groups were higher than that in the Ad-hBD3 group (Figure 7).

## Discussion

In periodontitis, the inflammatory microenvironments around the periodontal tissues could cause the absorption and destruction of alveolar bone (Preshaw and Bissett, 2019). How to reduce bone destruction and obtain bone regeneration in the inflammatory microenvironments has drawn much attention (Kong et al., 2013; Zhou et al., 2018). In our study, 1 μg/mL *E. coli*-LPS was chosen to create the inflammatory microenvironment according to a former study (Bindal et al., 2018). As a vital component of the periodontium, hPDLCs not only produce collagen but also produce mineralized tissue and have high levels of ALP activity. Some studies have found that hPDLCs can express bone-associated proteins, such as osteonectin, to form mineralized nodules, which means that they might potentially be osteogenic cells (Jonsson et al., 2011).

Antimicrobial peptides have been noted to play an essential role in maintaining the health status against disease in a fluctuant oral microenvironment (Pizzolato-Cezar et al., 2019; Vila et al., 2019). Human β-defensin 3 was shown to have broad-spectrum antimicrobial activity and also enhance the proliferation and osteogenic differentiation of hPDLCs (Wang et al., 2011; Zhou et al., 2018). However, synthetic recombinant hBD3 is too expensive to widely apply to disease treatment. In our former study, hPDLCs transfected by adenovirus containing the hBD3 gene were observed to express the hBD3 protein for at least 7 days. After transfection, Ad-hBD3-hPDLCs showed higher expression of osteogenic-related genes and proteins than normal hPDLCs in inflammatory microenvironments (Li et al., 2020).

Considering the potential synergistic therapeutic effects of modified AuNPs and adenovirus (Saini et al., 2006), Ad-hBD3-hPDLCs were then treated with AuNPs. TEM images display the cellular uptake of AuNPs (45 nm, 10 μM) in hPDLCs and their localization, mostly in autophagosomes and lysosomes. Another study explained the specific mechanism of AuNP uptake and the process through the endocytotic pathways (endosomes/lysosomes), revealing the intracellular diffusion coefficients as well as the characteristic transport velocities of endocytotic vesicles of AuNPs (Huefner et al., 2014). Viruses, as inherently structured nanoparticles, have many similar properties as nanomaterials, for example, nanoscale size and cellular uptake (Sirotkin et al., 2014). As a result, they can reveal many meaningful interdisciplinary applications. Viruses can form crystals, which are useful in generating long-range 3D ordered solids of nanostructured materials and can be used in X-ray systems (Falkner et al., 2005). Wang et al. (2003) found that AuNPs of 8–15 nm coupled to oligonucleotide detection probes could be used to detect hepatitis B and hepatitis C viruses in serum samples, which can be used to achieve the faster diagnosis of patients. In the field of gene therapy, a study showed that targeted delivery of AuNPs to the tumor tissue could be realized by a virus that expresses the receptor protein (Zharov et al., 2005). There are also some studies concerning the osteogenic induction effects of AuNP on various cells (Li et al., 2016; Xiang et al., 2018). In our study, Ad-hBD3 + AuNP treated hPDLCs showed the enhancement of osteogenic differentiation in inflammatory microenvironments, upregulated ALP activity, and promoted the mineralization of hPDLCs. This synergistic effect might be due to the enhancement effects of AuNPs in gene transfection. Rajesh et al. found that the gold nanoparticle-based targeted gene delivery system could target ovarian cancers caused by mutated p53 (Kotcherlakota et al., 2019).

The p38 mitogen-activated protein kinases (MAPKs) in mammals participate in various cellular activities involving proliferation, differentiation, and innate immunity. Yi et al., found that the p38 MAPK pathway can regulate the osteogenic differentiation of mesenchymal stem cells treated by AuNPs (Xiang et al., 2018). Wu et al. (2013) also indicated that p38 MAPK signaling plays a special role in the osteogenic differentiation of human periodontal ligament stem cells, which agrees with our results.

Furthermore, the results of *in vivo* experiments revealed the potential regeneration effects of AuNPs after Ad-hBD3 transfected rPDLCs. Former studies showed that the local transplantation of stem cells could promote tissue regeneration (Cooney et al., 2016; Li et al., 2018). In our study, after hBD3 gene transfection and AuNP treatment, rPDLCs showed some characteristics of stem cells, such as osteogenesis promotion effects, exactly as they did *in vitro*. Besides, the Ad-hBD3 + AuNPs group exhibited more powerful inhibiting effects of alveolar bone absorption than the Ad-hBD3 group, which indicates that modified AuNPs also worked *in vivo*. The immunohistochemical assay of p-p38 indicated that the p38 MAPK pathway might also be activated *in vivo* after adding AuNP into Ad-hBD3 transfected cells.

In conclusion, AuNPs combined hBD3 gene-modified hPDLCs could promote cell osteogenesis *in vitro* and reduce periodontal damage *in vivo*, and the p38 MAPK pathway might potentially regulate the process. Further studies are still needed to track transplanted cells *in vivo* to ensure that enough rhPDLCs was loaded. It is more rigorous if we used nude rats instead of normal rats to excluded the difference of immune response between individuals. We will continue to explore the more specific cellular mechanism of the relationship with AuNPs and gene transfection.

## Conclusion

Our study demonstrated the enhanced effect of osteogenic differentiation while applying AuNPs into Ad-hBD3-hPDLCs and uncovered its potential mechanism *via* the p38 MAPK pathway. The *in vivo* experiments illustrated the further effects of AuNPs on tissue engineering and periodontal regeneration after transplantation of Ad-hBD3-rPDLCs. These findings suggest that AuNPs might be promising nominees in genetic engineering technology with adenoviral vectors to achieve better therapeutic effects in different diseases.

## Data Availability Statement

The datasets presented in this study can be found in online repositories. The names of the repository/repositories and accession number(s) can be found below: GenBank, accession number BankIt2403139 B3180_hBD3(EE).seqMW305475.

## Ethics Statement

All animal experiments were approved by the Ethics Committee of Nanjing Stomatological Hospital, Medical School of Nanjing University. The animal experiments were conducted at the Nanjing Mergene Biotechnology Development Co., Ltd., (accreditation number: SYXK 2017-0066) according to the policies and guidelines for institutional animal care of Nanjing University, Nanjing, China.

## Author Contributions

LL and YZ wrote the manuscript. LL and JZ conducted the experiments. QZ and MW provided the valuable suggestions. WY, YL, and FY advised the work and modified the manuscript. All authors have approved the final version of the manuscript.

## Conflict of Interest

The authors declare that the research was conducted in the absence of any commercial or financial relationships that could be construed as a potential conflict of interest.
